# Graphical Tools for Comparative Genome Analysis

**DOI:** 10.1002/(SICI)1097-0061(200004)17:1<6::AID-YEA15>3.0.CO;2-V

**Published:** 2000

**Authors:** Jo Dicks

**Affiliations:** John Innes Centre Norwich Research Park Colney Norwich NR4 7UH UK

## Abstract

Visualization of data is important for many data-rich disciplines. In biology, where data sets are becoming larger and more complex, grephical analysis is felt to be ever more pertinent. Although some patterns and trends in data sets may only be determined by sophisticated computional analysis, viewing data by eye can provide us with an extraordinary amount of information in an instant. Recent advances in bioinformatic technologies allow us to link graphical tools to data sources with ease, so we can visualize our data sets dynamically. Here, an overview of graghical software tools for comparative genome analysis is given, showing that a range of simple tools can provide us with powerful view of the differences and similarities between genome.


**Yeast**
*Yeast* 2000; **17**: 6–15.

**Review Article**

### Graphical tools for comparative genome analysis

Jo Dicks*
*John Innes Centre, Norwich Research Park, Colney, Norwich NR4 7UH, UK*

*Correspondence to:
J. Dicks, John Innes Centre,
Norwich Research Park, Colney,
Norwich NR4 7UH, UK.
E-mail: jo.dicks@bbsrc.ac.uk*

Received: 1 February 2000
Accepted: 22 February 2000

### Abstract

**Visualization of data is important for many data-rich disciplines. In biology, where data sets are becoming larger and more complex, graphical analysis is felt to be ever more pertinent. Although some patterns and trends in data sets may only be determined by sophisticated computational analysis, viewing data by eye can provide us with an extraordinary amount of information in an instant. Recent advances in bioinformatic technologies allow us to link graphical tools to data sources with ease, so we can visualize our data sets dynamically. Here, an overview of graphical software tools for comparative genome analysis is given, showing that a range of simple tools can provide us with a powerful view of the differences and similarities between genomes. Copyright © 2000 John Wiley & Sons, Ltd.**

**Keywords:** **graphical tools; comparative genome analysis; Java**

### Introduction

In *Alice's Adventures in Wonderland* [1] Lewis Carroll wrote: 'What is the use of a book', thought Alice, 'without pictures or conversations?'. The alliance of biology and computer science has presented us with a large library of books in the form of databases, text files, spreadsheets and other types of data source. For many of these sources, it is difficult to make sense of the data without the use of graphics. One area of bioinformatics, data visualization, aims to add pictures to the books, and many of the groups involved in such projects are looking at ways to compare genomes, or parts of genomes, graphically. The scope of such comparisons can range from a tiny part of a genome, such as a gene sequence, to an overview of the relative arrangements of gross chromosomal segments throughout the genomes of two or more species.

In the last couple of years, Java has become the predominant computer programming language for graphical tools in the bioinformatics community. Java programmes are versatile, as they run on most types of computer and as they may also be run interactively within a World Wide Web (WWW) browser. The ability to link an interactive graphical tool to a data source such as a database and to access that tool within a WWW browser has opened up data analysis of biological information to a wider audience. It means that a user does not have to own expensive software, just a WWW browser, in order to gain access to a multitude of information and analysis tools. This revolution also questions the way that graphical tools are designed. We must consider whether we wish others to be able to use our software. This may have consequences on how we link our graphics to our data sources, whether our software is dependent or independent of a data source, and other factors such as the programming language we choose.

To illustrate the difference between software that is dependent on a data source and software that is independent of a data source, let us consider one of the first public graphical software tools for comparative genome analysis. In 1996 a series of graphical tools [2], centred on the Oxford Grid [3], was published. The graphics were written using the C programming language, such that they were integrated with the ACEDB database management system [4], depending directly on data held within the ACEDB database. Unlike many of the ACEDB graphical tools, the comparative mapping displays

Copyright © 2000 John Wiley & Sons, Ltd.

could not be accessed over the Internet using the WWW version of ACEDB. Consequently, a user would need to download ACEDB onto his/her own machine in order to view the displays, requiring a level of computer expertise that could not be expected of the casual user. Furthermore, the displays could only be used with data held within an ACEDB database, so a user would need to enter new data directly into his/her private copy of the database in order to display it. However, there were benefits to be gained by tightly linking the displays with the database management system. Many database curators, particularly those of the crop plant genomes, use the ACEDB system. These curators would be able to use the comparative mapping displays without changing their data format. Furthermore, the displays could all share the same data, so that moving between them, to gain different views of a comparison between the genomes of two species, was rapid and simple.

Figure 1 gives an example of the Oxford Grid, which illustrates the distribution an homologous loci between two species, here human and mouse. Rows and columns represent chromosomes, which may be drawn equally sized or relative to the size of the chromosome. Each dot represents an homology, such that a dot in the (14,12) cell denotes an homology between a locus on chromosome 14 in the first species (human) and a locus on chromosome 12 in the second (mouse). Large clusters of dots are indicative of conserved chromosomal segments. By clicking on a cell within an Oxford Grid and asking for a PairMap in the Oxford Grid's pull-down menu, the user then sees a more detailed comparison of the chromosomes involved in that cell. In our earlier example, this would be mean comparing locus order between chromosome 14 in species 1 and chromosome 12 in species 2, as shown in Figure 2. Note that a diagonal line in PairMap going from the left bottom hand corner to the right top hand corner would indicate a conserved chromosomal segment. A diagonal going the other way would indicate a segment that had become inverted in one of the species.

Indeed, the Oxford Grid is a popular general way to compare the distribution of elements between two species. It can also show paralogy data, a comparison of a species with itself, the within-species homologies arising as a result of duplication events. In addition, the elements it displays do not have to be genes. They could be any element that occurs along a chromosome. Other groups have recognized the value of the Oxford Grid and have wished to use it to display their data. However, they may have found that software that was written in C and that was tightly integrated with ACEDB was not suitable for their needs, as they used a different database management system or they needed to display the results within a WWW browser. Consequently, different versions of the Oxford Grid have arisen, notably a Java prototype developed in 1997 by the USDA Agricultural Genome Information Server (AGIS) group [5], to compare the genomes of crop plants, and a version written by the Jackson Laboratory [6] in Bar Harbor, Maine, to compare their mouse data with that of human.

There are two points to take into account here. The first is that the same graphics are useful for greatly different sets of species groups. Indeed, this should be a goal for designers of such software. Second, each group could have used their time much better had they been able to share a common piece of software. We have already seen that Java allows us to write software that can be used on most computer systems and within WWW browsers, so that most people can use it. Java tools can also be interfaced easily to many database management systems. Consequently, we have reached a point where it may be effective for a bioinformatics group to share their software with other groups. With this in mind a new, more generic version of the Oxford Grid has been written in Java. It is called the GridMap, and has the power to reproduce any of the four comparative mapping displays integrated within ACEDB, although with a slight loss of detail. Indeed GridMap, which we will discuss later, has been tested by another bioinformatics group and interfaced to their comparative genome database, with some success.

A major problem with comparative genome analysis is access to data. The value of a series of graphical tools is diminished if the data we wish to display lie in separate data sources that are not integrated in any way. However, software developers working on graphical software for comparative genome analysis are often working on, or closely with developers of, comparative mapping databases or distributed computer systems that collate comparative data from multiple data sources. In fact, this alliance between data production and the development of analysis tools is a common and highly productive phenomenon within

Copyright © 2000 John Wiley & Sons, Ltd.

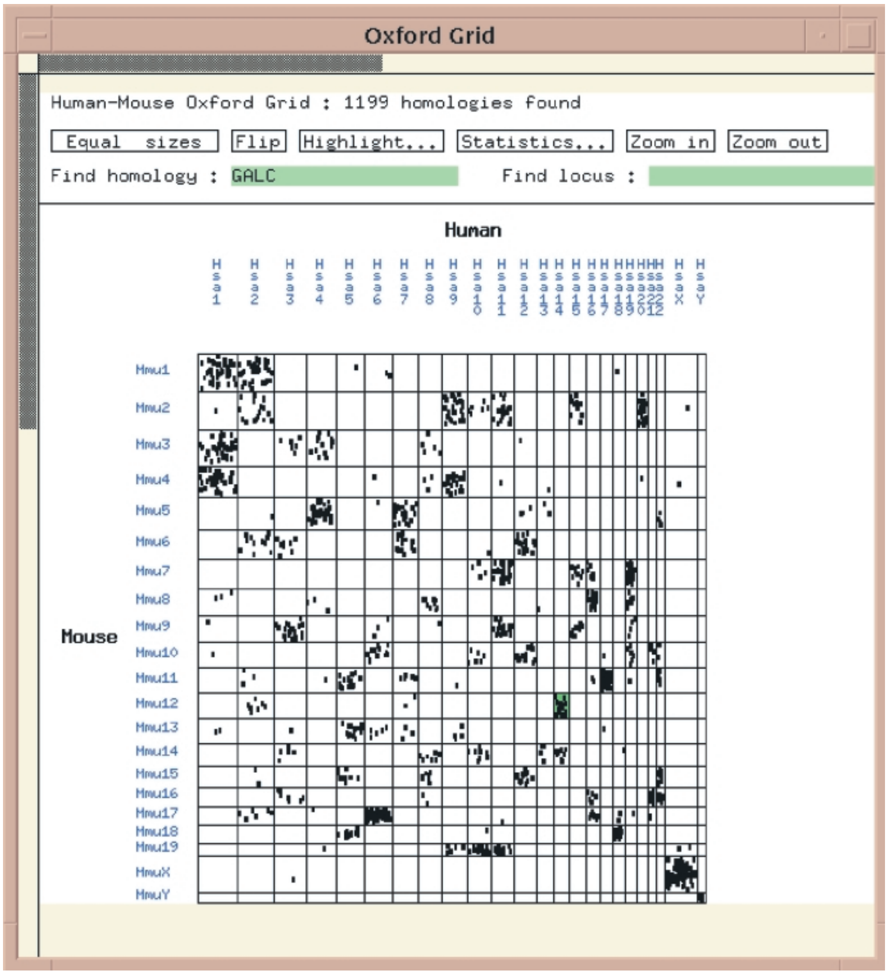

**Figure 1.** An Oxford Grid, showing the distribution of 1199 homologous loci between human and mouse. (Copyright © 1996 University of Oxford)

bioinformatics. However, despite a great deal of interest, comparative genome data sources have not arrived as quickly as expected, probably due to the unusually high amount of curator's time needed to create expertly-validated data sets. Consequently, the development of tools to compare genomes has been slower than many other types of tools. However, the quantity of tools available publicly is now great enough to compare genomes systematically and the coming years promise to increase this significantly.

### Graphical tools

The Bioinformatics Research Group [7] at the John Innes Centre (JIC) [8] specializes in comparative genome analysis, including the development of visualization tools for comparative data. The group is a member of the UK Crop Plant Bioinformatics Network (UK CropNet) [9], a BBSRC-funded consortium of six UK groups aimed at developing databases and software tools for the analysis of crop plant data, with a special emphasis on comparative genome analysis. A method to compare the arrangements of conserved chromosomal segments across several species simultaneously, well known within the crop plant community, is the Circular Genome Map, popularized in papers such as Moore *et al.* [10]. Figure 3 shows the Genome Map Viewer [11], a Java graphical tool recently developed by UK CropNet, which gives a computer-generated version of this diagram.

Copyright © 2000 John Wiley & Sons, Ltd. 

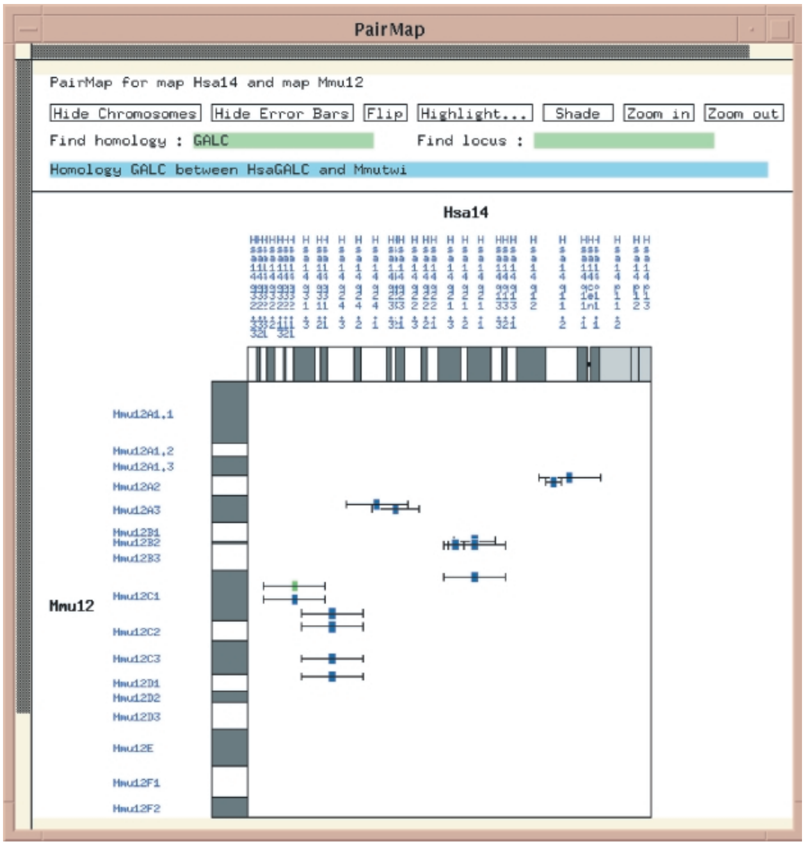

**Figure 2.** A PairMap showing the relative locations, with errors shown as bars, of homologous loci between human chromosome 14 and mouse chromosome 12. (Copyright © 1996 University of Oxford)

The Genome Map Viewer shown in Figure 3 depicts the genomes of three of the grasses, an economically important species group. The model grass is rice and we see that the chromosomes of rice are laid end-to-end and circularized, forming the innermost red circle of this display. Currently, there are thought to be as few as 31 conserved chromosomal segments within the grasses (Katrien Devos, personal communication). These segments have similar gene content and order, although small rearrangement events may have taken place within them. The segments are named after their location in rice. For example, rice chromosome 1 contains segments R1a and R1b, and rice chromosome 4 contains R4a, R4b, R4c and R4d. The relative arrangements of these segments may be seen in the outer concentric circles. For instance, the yellow circle represents the chromosomes of foxtail millet. It is easy to see that foxtail millet chromosome IV consists of the same segments as that of rice chromosome 6 (R6a, R6a, R6c and R6d) and that these are in the same order in each species. However, the chromosomes do not always have such a clear one-to-one relationship with each other. For example, foxtail millet chromosome II consists of the segments R7a, R9 and R7b, such that the loci located on rice chromosome 9 have been translocated into the middle of rice chromosome 7. This translocation is denoted by a blue arc drawn just outside the segments, its arrowhead denoting the point of insertion. Note that this insertion point is also seen in sugarcane. Common rearrangement events such as this give us clues as to the phylogenetic relationship of the grasses, inferring that such events occurred in a common ancestor of the species that share them.

The Genome Map Viewer can also display elements such as loci. A feature of the Circular Genome Map is that loci that lie on a line drawn outwards from the centre of the map, such that it

Copyright © 2000 John Wiley & Sons, Ltd. 

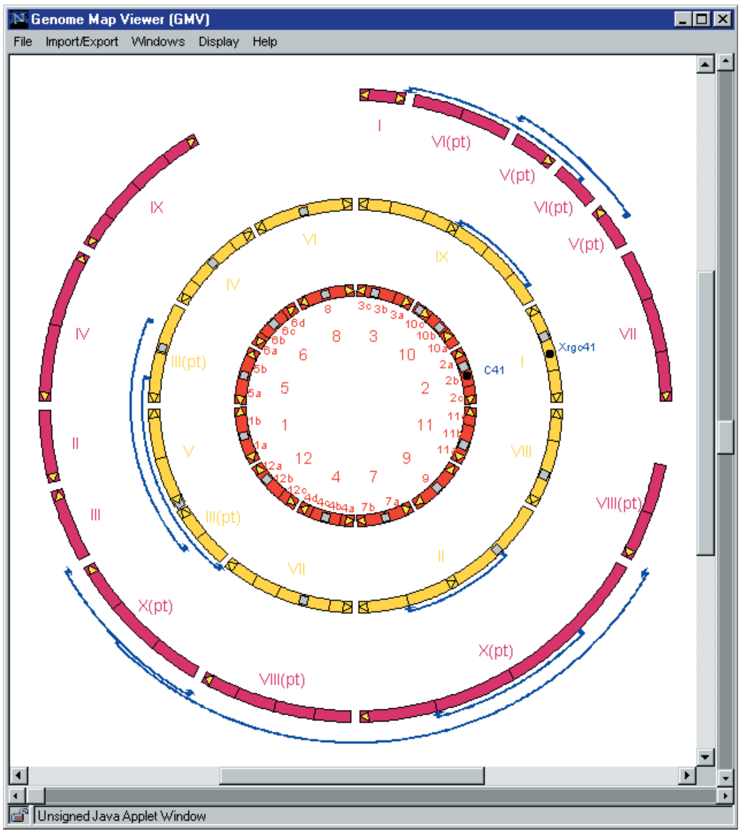

**Figure 3.** A Genome Map Viewer, showing the distribution of conserved chromosomal segments between rice (innermost circle), foxtail millet (middle circle) and sugarcane (outermost circle). (Copyright © 1996–2000 UK Crop Plant Bioinformatics Network (UK CropNet))

bisects each concentric circle at an equal angle, are usually homologous to one another. For example, Figure 3 shows the locus C41 on rice chromosome 2, lying within segment R2b. We also see a locus Xrgc41 on foxtail millet chromosome 1, also within segment R2b. Indeed, these two loci are known to be homologues. Thus, the map gives a simple way to identify potential locations of unmapped genes, based on the locations of genes in other species that are similar in some way. The Circular Genome Map has been used extensively for crop plant species. However, it should be applicable to other species groups. The Genome Map Viewer software is freely available and may be downloaded by any interested groups, together with documentation on how to interface the tool to particular types of data source.

The GridMap [12] is a generic grid-drawing Java tool that is particularly useful for the display of comparative genome data. However, its generic design enables it to display gridded data vastly different to biological information, such as railway timetables, as long as the data conform to a particular but simple tabular format. In fact, GridMap does not 'understand' biological concepts but it is very good at displaying biological data. Figure 4 shows it being used as an Oxford Grid, comparing the distribution of homologous loci between rice and maize. Figure 5 shows it being used to compare two short DNA sequences. Indeed, its scope extends to non-comparative biological uses. At JIC it has recently been used as a tool to verify locus order on newly made genetic maps.

The UK CropNet group at the Institute of Grassland and Environmental Research (IGER) [13] has produced two related Java graphical tools for the comparison of linear maps. The first of these, the Comparative Physical and Genetic Map (CPGMap) [14] was written to compare gene order for physical and genetic maps of the same chromosome in the same species. However, it can also be

Copyright © 2000 John Wiley & Sons, Ltd. 

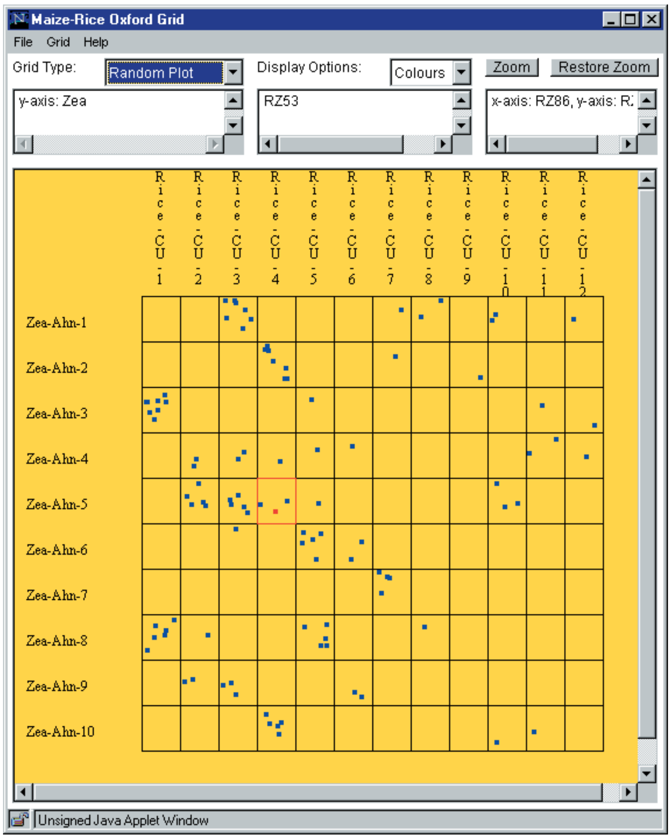

**Figure 4.** A GridMap, here used as an Oxford Grid of rice and maize. (Copyright © 1996–2000 UK Crop Plant Bioinformatics Network (UK CropNet))

used to compare maps between species. Figure 6 shows an example of a CPGMap, where we see a comparison of locus order between physical and recombinant inbred (RI) maps of *Arabidopsis thaliana* chromosomes IV and V. The second graphical tool, the Pairwise Comparative Map (PCM) [15], was written to compare maps from different species. Figure 7 shows an example of a PCM, comparing linkage groups of two closely related dicots, *Brassica napus* and *Brassica oleracea.*

The UK CropNet and JIC graphical tools have been interfaced to text files and to the UK CropNet comparative database ComapDB [16] using CORBA, a specification that allows entities such as databases and analysis tools to talk to one another over the Internet. ComapDB uses the ACEDB database software, which now comes with a recently developed CORBA interface called CITA [17]. With CORBA it is possible to write graphical software interfaced to database management systems such as ACEDB, but in such a way that other groups, who may have no interest in ACEDB, can adopt the software for themselves without having to rewrite it. This breaking down of systems into its constituent parts, componentization, is revolutionizing bioinformatics and, if used well, can greatly increase productivity within groups.

The UK Animal Bioinformatics Network [18] is a BBSRC-funded network for database and software development for animal data. Its main site is the Roslin Institute, Edinburgh [19] and it is the sister network to UK CropNet. The Roslin bioinformatics group is currently writing a Java version of their Anubis map display tool to compare linear chromosomal segments across a number of species. In contrast to the plant community, the animal mapping community uses linear maps rather than circular maps to compare genomes. The new Anubis display will eventually replace the existing

Copyright © 2000 John Wiley & Sons, Ltd.

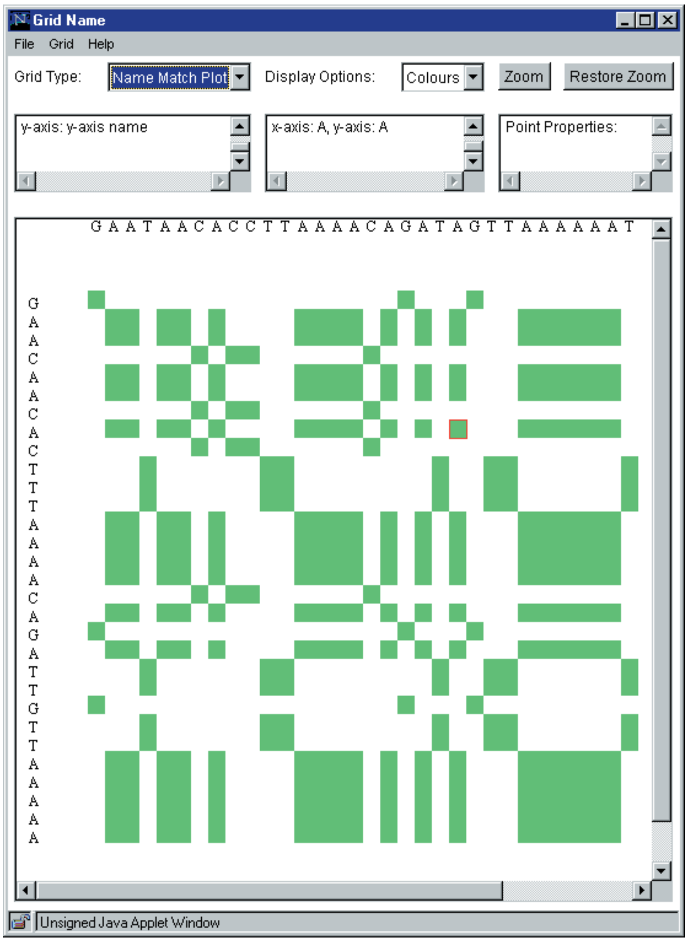

**Figure 5.** A GridMap, here used to compare two DNA sequences. (Copyright © 1996–2000 UK Crop Plant Bioinformatics Network (UK CropNet))

tool [20] shown in Figure 8. It will display the same kind of information as seen in a pie-shaped segment of the Circular Genome Map but in more detail. Thus the tool will be highly important to both UK bioinformatics networks.

A recently developed tool for comparing linear maps across different species is the Comparative Genome Mapping Tool (CGMT) [21] from the National Center for Genome Research (NCGR) in Santa Fe, New Mexico [22]. NCGR's tool compares two linear maps drawn in separate windows, on the criteria of common RFLP probe, name or sequence similarity. The two maps are able to communicate with one another, and to highlight homologous structures across the two, making clear the relationships between them.

Although graphical tools for the high-level comparison of whole genomes are a relatively recent phenomenon, tools for low-level comparisons of elements such as DNA sequences are more prevalent. It is likely that their development has gone hand-in-hand with the development of computational methods for sequence analysis, such as phylogenetic methods. For example, Alfresco [23] compares multiple sequences from putatively homologous regions in different species. Results from various analysis tools, such as gene prediction, protein homology and regulatory sequence prediction programmes are visualized and used to find corresponding sequence domains. Tools such as CINEMA [24] and Jalview [25] are used for the visualization and manipulation of multiple sequence alignments of DNA and protein sequences.

Other groups are currently highlighting compara-

Copyright © 2000 John Wiley & Sons, Ltd.

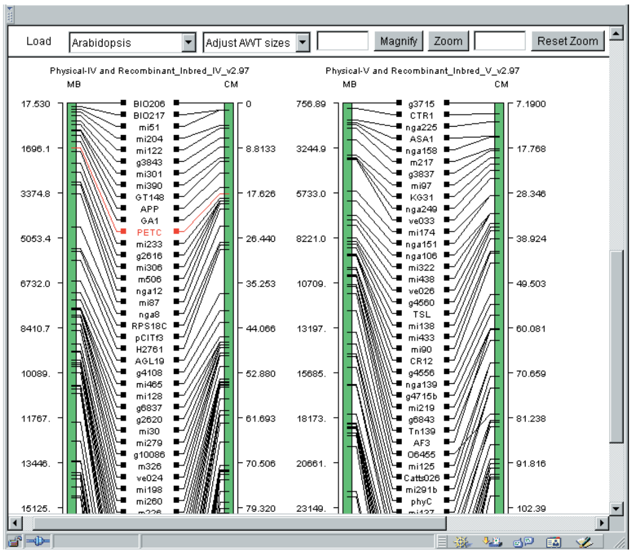

**Figure 6.** A Comparative Physical and Genetic Map (CPGMap), showing physical and RI maps for *Arabidopsis thaliana* chromosomes IV and V. (Copyright © 1996–2000 UK Crop Plant Bioinformatics Network (UK CropNet))

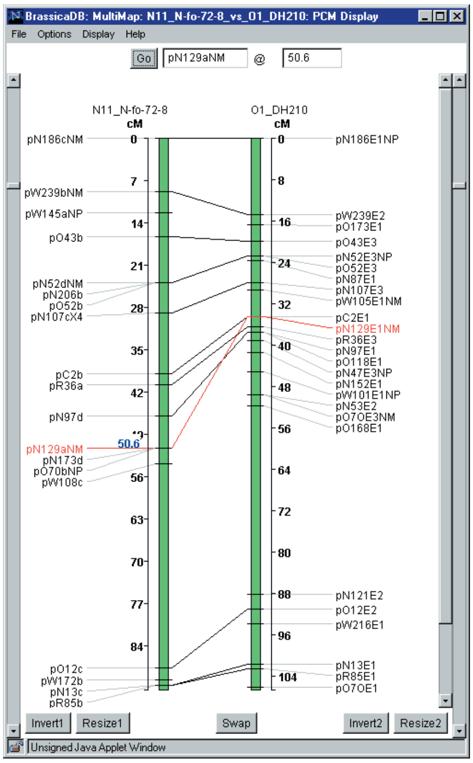

**Figure 7.** A Pairwise Comparative Map (PCM), comparing locus order of *Brassica napus* linkage group 11 to that of *Brassica oleracea* linkage group 1. (Copyright © 1996–2000 UK Crop Plant Bioinformatics Network (UK CropNet))

tive genome analysis as an important area of research. The recently established USDA-ARS Center for Bioinformatics and Comparative Genomics (CBCG) [26] at Cornell University is planning a series of tools for such analysis. CBCG is the prime US bioinformatics development group for crop plants following the end of the AGIS service. CBCG has a close relationship with bioinformatics networks such as UK CropNet, and coordinated development will be a priority. The next few years promise to provide a wealth of tools for the graphical analysis of comparative genomic data.

### Conclusions

We have seen that there is a growing number of tools for the comparison of biological elements, both within and between species. These tools range from low-level analyses of DNA sequences to high-level comparisons of whole genomes. It is noticeable that a recent lack of tools for whole-genome comparison has been mirrored by a lack of computational methods for the same data and, indeed, for comprehensive public databases for comparative data. However, major comparative genome databases are in development and we can soon expect tools with which to analyse the data. Furthermore, the shift towards component development within the bioinformatics community pro-

Copyright © 2000 John Wiley & Sons, Ltd. 

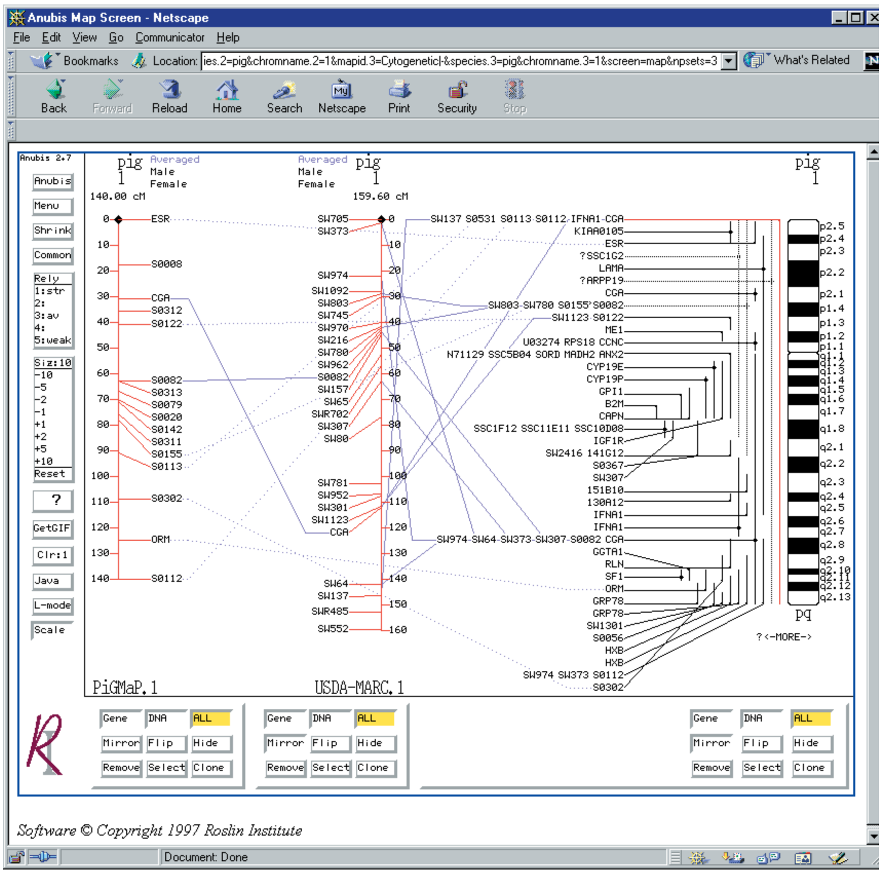

**Figure 8.** The Anubis comparative mapping tool, showing a comparison of the Cytogenetic, PiGMap.1 (sex avg) and USDA-MARC.1 (sex avg) maps for pig chromosome 1. (Copyright © 1997 Roslin Institute)

mises to provide versatile graphical tools that can be interfaced to multiple data sources, can be tailored to many different species groups, and can be deployed across the Internet in WWW browsers, as well as on a user's machine. If this trend continues, developers can concentrate on writing tools that have yet to be developed rather than reproducing existing tools.

### Acknowledgements

I would like to thank Professor J. H. Edwards for introducing me to the value of comparative genome analysis and to the MRC for supporting early work in this area. For more recent work, I would like to thank the BBSRC for their continued support of UK CropNet. Thank you to my colleagues, particularly my fellow UK CropNet developers and my US crop plant collaborators, for their hard work in producing tools that we hope will benefit the wider bioinformatics community.

### References

1. Carroll L. 1865. *Alice's Adventures in Wonderland.* Macmillan and Co., London.
2. Dicks J. 1997. The display of comparative mapping data using ACEDB. *Genetic Mapping of Disease Genes*, Pawlowitski I-H, Edwards JH, Thompson EA (eds). Academic Press, London: 221–235.
3. Edwards JH. 1991. The Oxford Grid. *Ann Hum Genet* 1991; **55**: 17–31.
4. Durbin R, Thierry-Mieg J. 1991. ACEDB. **http://www.acedb.org/**
5. Agricultural Genome Information Server. **http://probe.nalusda.gov:8000/**
6. Jackson Laboratory. **http://www.jax.org/**
7. John Innes Centre Bioinformatics Research Group. **http://jiio8.jic.bbsrc.ac.uk/**
8. John Innes Centre. **http://www.jic.bbsrc.ac.uk/**
9. UK Crop Plant Bioinformatics Network. **http://synteny.nott.ac.uk/**
10. Moore G, Devos KM, Wang Z, Gale MD. 1995. Grasses, line up and form a circle. *Curr Biol* **5**: 737–739.

Copyright © 2000 John Wiley & Sons, Ltd. 

11. Davenport GF, O'Malia AD, Dicks JL. 1999. The Genome Map Viewer. **http://synteny.nott.ac.uk/software.html**
12. Priestly MH, Dickson JS, Dicks JL. 1999. GridMap III. **http://synteny.nott.ac.uk/software.html**
13. Institute of Grassland and Environmental Research. **http://www.iger.bbsrc.ac.uk/igerweb/**
14. Dickson JS. 1998. The Comparative Physical and Genetic Map. **http://synteny.nott.ac.uk/software/cpg/cpg-top.htm**
15. Dickson JS. 1998. The Pairwise Comparative Map. **http://synteny.nott.ac.uk/pairwise/Welcome.html**
16. Davenport GF, Dicks JL. 1999. protoComapDB—prototype for a comparative mapping database. **http://jiio8.jic.bbsrc.ac.uk/bioinformatics-research/protoComapDB/index.html**
17. McWilliam H. 1999. CORBA Interface To ACEDB. **http://jiio10.jic.bbsrc.ac.uk/development/CITA/**
18. UK Animal Bioinformatics Network. **http://www.ri.bbsrc.ac.uk/bioinformatics/databases.html**
19. Roslin Institute. **http://www.ri8.8bbsrc.ac.uk/**
20. Mungall C. 1997. Anubis. **http://www.ri.bbsr4c4.ac.uk/anubis/**
21. Pecherer RM, Polacco M, Zhang HB, Beavis WD, Cartner T, Shroeder S. 1999. Comparative Genome Mapping Tool. **http://www.ncgr.org/research/cgmt/**
22. National Center for Genome Research. **http://www.ncgr.org/**
23. Jareborg N. 1998. Alfresco. **http://www.sanger.ac.uk/Software/Alfresco/**
24. Attwood TK, Payne AWR, Michie AD, Parry-Smith DJ. 1997. A Colour INteractive Editor for Multiple Alignments—CINEMA. **http://www.biochem.ucl.ac.uk/bsm/dbbrowser/CINEMA2.1/**
25. Clamp M, Cuff J, Barton G. 1998. Jalview—Analysis and Manipulation of Multiple Sequence Alignments. **http://www2.ebi.ac.uk/embnet.news/vol5_4/embnet/body_jalview.html**
26. USDA-ARS Center for Bioinformatics and Comparative Genomics. **http://genome.cornell.edu/**

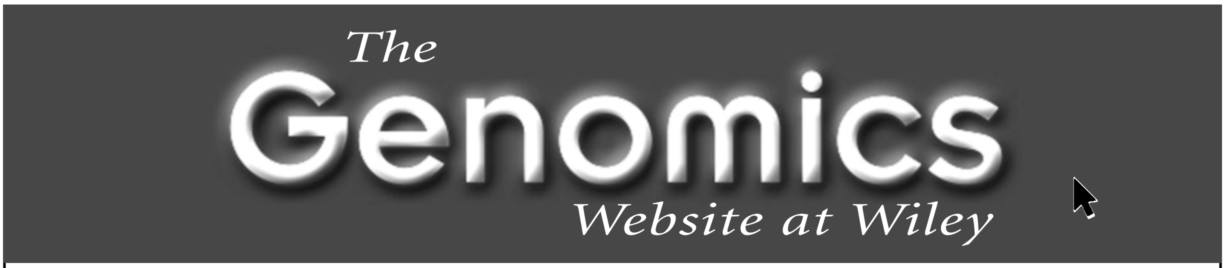

**www.wiley.co.uk/genomics**

The **Genomics** website at Wiley is a new and **DYNAMIC** resource for the genomics community, offering **FREE** special feature articles and new information **EACH MONTH**.

Find out more about *Comparative and Functional Genomics*, and enter the **Free Prize Draw** for the chance to win a year's free subscription.

Visit the **Library** for hot books in Genomics, Bioinformatics, Molecular Genetics and more.

Click on **Primary Research** for information on all our up-to-the minute journals, including: *Genesis, Bioessays, Gene Function and Disease*, and the *Journal of Gene Medicine*.

Let the **Genomics** website at Wiley be your **guide** to genomics-related web sites, manufacturers and suppliers, and a calendar of conferences.

WILEY

1516

Copyright © 2000 John Wiley & Sons, Ltd. 

